# Morphological patterns of anaemia among pregnant women from Sudan

**DOI:** 10.4102/ajlm.v8i1.743

**Published:** 2019-10-14

**Authors:** Abuobieda B. Abusharib

**Affiliations:** 1 Department of Pathology, College of Medicine, Najran University, Najran, Saudi Arabia

**Keywords:** anaemia, pregnancy, morphological, pattern, clinicopathological

## Abstract

**Background:**

Morphological patterns of anaemia in pregnancy are considered essential for classification, diagnosis and management of patients, especially in regions with high maternal mortality like Sudan.

**Objectives:**

This study evaluated morphological patterns of anaemia among pregnant women in Sudan and morphological differences across characteristics of participants.

**Methods:**

This cross-sectional study was conducted from September 2016 to February 2017. A total of 200 women were selected according to specific criteria. Laboratory tests were performed for complete blood count, blood smears were performed for morphology and vitamin B12, folate and iron levels were measured. Participants were classified as: normochromic normocytic, microcytic hypochromic, macrocytic or dimorphic. Further classification based on haemoglobin levels was also performed.

**Results:**

A total of 116 participants (58%) had a dimorphic pattern, followed by 50 participants (25%) with a microcytic hypochromic pattern, 20 participants (10%) with a macrocytic pattern and 14 participants (7%) with a normochromic normocytic pattern. Participants with the dimorphic pattern also had low levels of iron and folate. The majority of dimorphic participants presented with mild anaemia, whereas the majority of participants with the microcytic hypochromic pattern presented with moderate or severe anaemia. A high percentage of participants in late pregnancy had the dimorphic pattern, and there were significant differences in the degree of anaemia by parity, gestational age and regular intake of haematinic supplements.

**Conclusion:**

The most frequent morphological pattern of anaemia in this study was dimorphic, followed by microcytic hypochromic, macrocytic and normochromic patterns. Morphological patterns appeared to predict types of vitamin and mineral deficiency and the degree of anaemia.

## Introduction

Anaemia affects more than 55% of women in Sudan; most of them are pregnant.^1^ According to World Health Organization (WHO) guidelines, anaemia in pregnancy is defined as a haemoglobin level under 11 gm/dL for the first and third trimester and under 10.5 gm/dL for the second trimester.^2,3,4^ Both red blood cell (RBC) mass and plasma volume expand in pregnancy, reaching the maximum level in the second trimester. However, the expansion of 35% – 40% in plasma volume exceeds the 20% – 25% increase in RBC mass; as a result, there is a dilutional drop in haemoglobin concentration, haematocrit and RBC count. Additionally, there is a 2- to 3-fold increase in iron requirements and 10- to 20-fold increase in folate requirements.^5,6^

In Africa, nutritional deficiency is a common cause of anaemia. Three factors contribute to the pathogenesis of vitamin and mineral deficiencies in pregnancy. Firstly, both the growing foetus and maternal tissues use the entire maternal stores of minerals and vitamins (i.e. there is an increase in demand). Secondly, in developing countries, there is a lack of vitamin and mineral supplement use or inadequate food intake during pregnancy. Thirdly, folic acid and vitamin B12 absorption are usually impaired during pregnancy. Infectious diseases, such as malaria, helminths and HIV, besides poor quality of health services, poverty and ignorance, indirectly participate in the aetiology of anaemia in pregnancy.^5^

Anaemia during pregnancy is one of the leading causes of maternal and foetal morbidity and mortality in almost all developing countries.^6^ Even moderate bleeding in an anaemic pregnant woman could be a risk for preterm delivery. Moreover, foetal growth restriction and low birth weight are increased when haemoglobin drops. Also, maternal anaemia adversely affects cognitive, behavioural and physical development in infants. Anaemia also depresses immune status and increases morbidity from infections in neonates. In 2011, the WHO published a new classification for anaemia in pregnancy based on haemoglobin concentration and gestational age as follows: in mild anaemia, haemoglobin concentration is 10.00–10.99 g/dL for the first and third trimester, and 10.49–10.99 g/dL for the second trimester. In moderate anaemia, the haemoglobin concentration is 7.00–9.99 g/dL regardless of trimester, whereas in severe anaemia, the haemoglobin concentration is less than 7.00g/dL irrespective of trimester.^6^

Based on the morphology of RBCs and blood cell indices, anaemia is classified into normocytic normochromic, microcytic hypochromic, macrocytic and dimorphic anaemia. Each type suggests specific aetiological factors, so an evaluation of the morphology of RBCs and clinical features among pregnant women could help in the diagnosis and management of patients. However, there is little research on the characteristic morphology of RBCs in anaemic pregnant women in Sudan.^1,7^ The objective of this study was to evaluate the morphology of RBCs among anaemic pregnant women and examine differences in morphological features across some clinical characteristics of study participants.

## Methods

### Ethical considerations

Khartoum North Teaching Hospital Research Committee issued an ethical approval for conducting this study (study approval number: 14/2016). Written consent was obtained from the participants, and a high level of protection was taken to maintain confidentiality of the collected data and usages for the study only.

### Study design

This was a cross-sectional study conducted at Khartoum North Teaching Hospital in Sudan from September 2016 to February 2017. Two hundred anaemic, pregnant women attending antenatal clinics in the hospital were included, after fulfilling specific selection and exclusion criteria. To be included in the study, participants had to be anaemic (according to WHO 2001 classification of anaemia in pregnancy) and have no history of recent transfusions, bleeding or chronic disease.

### Data collection

A questionnaire was designed to collect information on age, parity (primigravida: first pregnancy, multigravida: 2–4 pregnancies, or grand multipara: ≥ 5 pregnancies), gestational age (first trimester, second trimester, third trimester), level of education (illiterate, did not complete primary school, completed primary school, or above primary school) and economic status (annual income in US dollars, very poor: < 700, poor: 700–1400, enough: 1400–2800, good: > 2800), in addition to marital status (married, separated, or divorced), past medical history, haematinic supplement use (yes or no) and other obstetrical information. Furthermore, laboratory data were collected, including complete blood count, RBC morphology, iron profile, RBC folate and serum vitamin B12 level.

#### Statistical analysis

The collected data were transferred to the Statistical Package for Social Sciences (SPSS) computer program (IBM® SPSS® Statistics 19; IBM Company, Philadelphia, Pennsylvania, United States). The mean age ± standard deviation, distribution and frequencies of the characteristics were calculated, in addition to differences in the severity of anaemia among morphological patterns, using the chi-squared test. *p*-values less than 0.05 were considered significant for differences in the degree of anaemia among categories of parity, gestational age, haematinic levels and regular intake of haematinic supplements.

### Laboratory analysis

Nine mL of venous blood was collected and divided into three containers: ethylene diamine tetraacetic acid (EDTA), lithium heparin and clear containers. Roche/Hitachi Cobas systems (Cobas 6000 Analyzer series, 2012 June; Roche Diagnostics Corporation, Indianapolis, Indiana, United States) were used for measuring serum iron, iron binding capacity, serum ferritin, serum vitamin B12 and RBC folate. A quality control protocol was implemented, using normal, low and high range control sera. Additionally, complete blood count was measured using Sysmex KX20 automated analyser, manufacturer by Sysmex Corporation, September 2015 (Bellport, New York, United States), and direct peripheral smears were taken to be compared with Ethylene diamine tetraacetic acid (EDTA) smears; both types of smears were stained with Leishman’s stain and examined under the microscope for RBC morphological classification.

### Classifications of the study

Based on complete blood count parameters, mean corpuscular volume (MCV), mean corpuscular haemoglobin (MCH) and mean corpuscular haemoglobin concentration (MCHC) and their correlation with the morphology of smears in which the size of RBCs was compared to the nucleus of lymphocytes, the participants were grouped into four patterns of anaemia: microcytic hypochromic, macrocytic, normocytic normochromic and dimorphic (mixed macrocytic and microcytic). Also, both red cell distribution width and MCV were compared to RBC morphology. The normal reference range was 11% – 15% for red cell distribution width, 76–96 fl for MCV, 27–33 picograms for MCH and 33–36 g/dL for MCHC. Consequently, MCV less than 76 fl suggested microcytosis and (low MCV, low MCH and/or MCHC) indicated a microcytic hypochromic pattern, confirmed by morphology, while MCV greater than 96 fl with normal or elevated MCHC suggested macrocytic pattern, also confirmed by morphology. However, normal MCV, MCH and MCHC indicated a normocytic normochromic pattern, confirmed by morphology. The dimorphic pattern was suggested by the presence of mixed macrocytic and microcytic pattern in morphology and dual population in RBCs histogram, while MCV could be normal, low or high.

Participants were also categorised as having mild, moderate or severe anaemia, according to WHO guidelines for haemoglobin concentration in pregnancy. In mild anaemia, haemoglobin concentration was 10.00–10.99 g/dL for the first and third trimesters and 10.49–10.99 g/dL for the second trimester. In moderate anaemia, the haemoglobin concentration was 7.00–9.99 g/dL regardless of trimester. Similarly, in severe anaemia, the haemoglobin concentration was less than 7.00 g/dL irrespective of trimester. To simplify the analysis across some variables, participants were further categorised by considering participants with moderate or severe anaemia in one group, participants who were multigravidas or grand multiparas in another group and second or third trimester participants in a third group.

## Results

Participants’ mean age (± standard deviation) was 28.5 years (± 7.1 weeks), and the age range was 17–46 years, 44% of participants had not completed primary school and 39% were illiterate, 25% had very poor income and 60% had poor income (Table 1).

**TABLE 1:** Distribution of socio-demographic and clinical variables, Sudan, September 2016 to February 2017.

%	*N*	Socio-demographic and clinical variables
**Marital status**		
Married	150	75
Separated	32	16
Divorced	18	9
**Income**		
Very poor (< 700 dollars/year)	50	25
Poor (700–1400 dollars/year)	120	60
Enough (1400–2800 dollars/year)	26	13
Good (> 2800 dollars/year)	4	2
**Parity**		
Primigravida	36	18
Multigravida	134	67
Grand multipara	30	15
**Educational level**		
Illiterate	78	39
Did not complete primary school	88	44
Completed primary school	22	11
Above primary school	12	6
**Gestational age**		
First trimester	14	7
Second trimester	30	15
Third trimester	156	78

### Morphological patterns of anaemia

**FIGURE 1:**
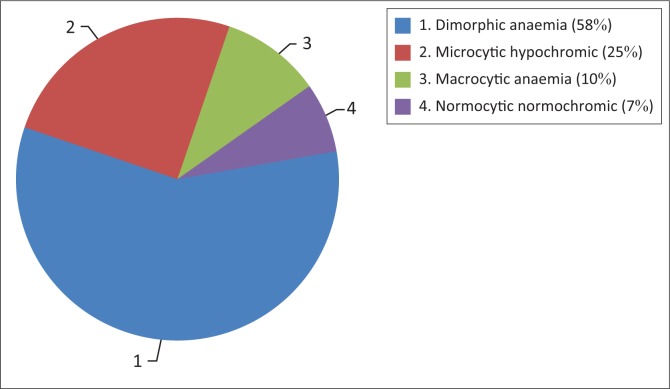
Distribution of morphological patterns of anaemia, Sudan, September 2016 to February 2017.

**FIGURE 2:**
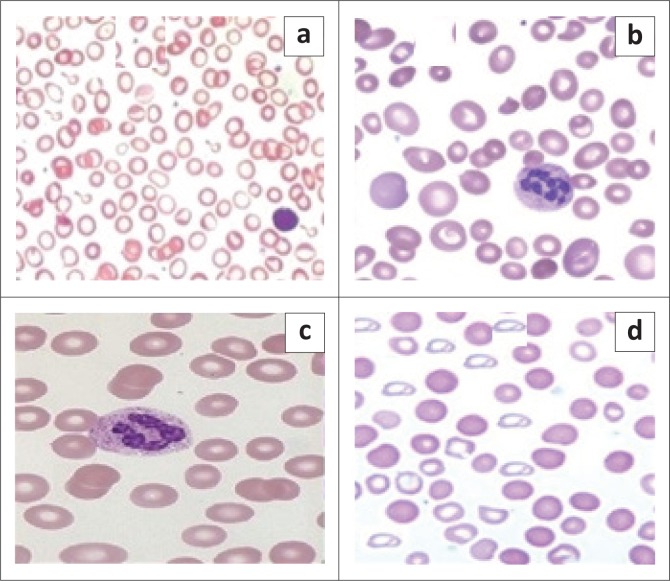
Photos of morphological patterns of anaemia, Sudan, September 2016 to February 2017. (a) Hypochromic microcytic, (b) macrocytic pattern, (c) normochromic normocytic and (d) dimorphic pattern.

Based on morphology and blood cell indices, 116 (58%) participants had the dimorphic pattern, 50 (25%) had the microcytic hypochromic pattern, 20 (10%) had the macrocytic pattern and 14 (7%) had the normocytic normochromic pattern (Figures 1 and 2).

### Dimorphic (microcytic and macrocytic) pattern

Representing 116 participants (58%), dimorphic was the most common morphological pattern of anaemia in our study; 74 participants (63.8%) had mild and 36 (31%) had moderate anaemia, and only 6 (5.2%) had severe anaemia. Moreover, 76 participants (65.6%) with the dimorphic pattern were multigravidas, and only 20 participants (17.2%) were primigravidas. The majority of participants (102; 88%) were in their third trimester. In this group, 96 participants (82.8%) had a low iron profile, 90 (77.6%) had low folate and 16 (13.8%) had low vitamin B12 levels (Tables 2 and 3).

**TABLE 2:** Morphological patterns of anaemia across participant characteristics, Sudan, September 2016 to February 2017.

Characteristics	Dimorphic (*N* = 116)		Microcytic hypochromic (*N* = 50)		Macrocytic (*N* = 20)		Normocytic normochromic (*N* = 14)	
	*N*	%	*N*	%	*N*	%	*N*	%
**Degree of anaemia**								
Mild	74	63.8	26	52.0	16	80.0	14	100.0
Moderate	36	31.0	20	40.0	4	20.0	0	0.0
Severe	6	5.2	4	8.0	0	0.0	0	0.0
Total (*N* = 200)	116	100.0	50	100.0	20	100.0	14	100.0
**Parity**								
Primigravida	20	17.2	12	24.0	4	20.0	6	42.8
Multigravida	76	65.6	28	56.0	16	80.0	8	57.2
Grand multipara	20	17.2	10	20.0	0	0.0	0	0.0
Total (*N* = 200)	116	100.0	50	100.0	20	100.0	14	100.0
**Gestational age**								
First trimester	4	3.4	10	20.0	0	0.0	0	0.0
Second trimester	10	8.6	16	32.0	0	0.0	4	28.5
Third trimester	102	88.0	24	48.0	20	100.0	10	71.5
Total (*N* = 200)	116	100.0	50	100.0	20	100.0	14	100.0
**Taking supplements**								
Yes	16	13.8	0	0.0	0	0.0	14	100.0
No	100	86.2	50	100.0	20	100.0	0	0.0
Total (*N* = 200)	116	100.0	50	100.0	20	100.0	14	100.0

**TABLE 3:** Difference in morphological patterns of anaemia with iron profile, folate, vitamin B12 levels, Sudan, September 2016 to February 2017.

Characteristics	Dimorphic (*N* = 116)		Hypochromic microcytic (*N* = 50)		Macrocytic (*N* = 20)		Normochromic normocytic (*N* = 14)	
	*N*	%	*N*	%	*N*	%	*N*	%
**Iron profile**								
Normal	20	17.2	0	0.0	20	100.0	14	100.0
Low	96	82.8	50	100.0	0	0.0	0	0.0
**Red cell folate**								
Normal	26	22.4	50	100.0	4	20.0	14	100.0
Low	90	77.6	0	0.0	16	80.0	0	0.0
**Serum B12**								
Normal	100	86.2	50	100.0	6	30.0	14	100.0
Low	16	13.8	0	0.0	14	70.0	0	0.0

### Microcytic hypochromic pattern

The microcytic hypochromic morphological pattern of anaemia was the second most frequent with 50 participants (25%). Of them, 26 (52%) had mild anaemia and 20 (40%) had moderate anaemia. The distribution of parity in this pattern revealed 28 (56%) were multigravidas, 10 (20%) were grand multiparas, and only 12 (24%) were primigravidas. Additionally, 24 (48%) participants were in their last trimester. None of the participants with this pattern had taken routine haematinic supplements during pregnancy. All participants with this pattern had a low iron profile, normal RBC folate and normal serum vitamin B12 (Tables 2 and 3).

### Macrocytic pattern

The macrocytic pattern was the third most common, representing 20 participants (10%). All 20 were in the third trimester and 16 of them (80%) had mild anaemia. None had taken routine mineral or vitamin supplements during their pregnancy (Table 2). Fourteen (70%) had low vitamin B12 and 16 (80%) had low folate levels (Tables 2 and 3).

### Normocytic normochromic pattern

The normocytic pattern was the least common, with 14 participants (7%). All were regularly taking mineral and vitamin supplements. All participants had only mild anaemia. Moreover, all had a normal iron profile, normal serum vitamin B12 and normal RBC folate levels (Tables 2 and 3).

### Degree of anaemia across variables

Based on the haemoglobin level, 130 participants (65%) had mild anaemia, 60 (30%) had moderate anaemia and 10 (5%) had severe anaemia (Table 4). A total of 96 participants (73.9%) in the mild anaemia group presented in the second or third trimester, whereas 34 (26.1%) participants were in the first trimester. However, 60 participants (85.7%) in the moderate or severe anaemia group were in the second or third trimester, and 10 (14.3%) participants were in the first trimester; the difference between the two groups was significant (*p* = 0.025). Analysis of the degree of anaemia and parity showed 100 participants (76.9%) in the mild anaemia group were multigravidas or grand multiparas, whereas 30 participants (23.1%) were primigravidas. However, 64 participants (91.4%) in the moderate or severe anaemia group were multigravidas or grand multiparas, and only 6 participants (8.6%) were primigravidas; the difference between the two groups was significant (*p* = 0.034).

**TABLE 4:** Differences in the degree of anaemia with gestational age, parity and haematinic supplement intake, Sudan, September 2016 to February 2017.

Characteristics	Mild anaemia		Moderate or severe anaemia		*p-*value
	*N*	%	*N*	%	
**Gestational age**					
First trimester	34	26.1	10	14.3	0.025
Second or third trimester	96	73.9	60	85.7	-
Total	130	100.00	70	100.0	-
**Parity**					
Primigravida	30	23.1	6	8.6	0.034
Multigravida or grand multipara	100	76.9	64	91.4	-
Total	130	100.00	70	100.0	-
**Haematinic supplement intake**					
Yes	26	20.00	4	5.7	0.026
No	104	80.00	66	94.3	-
Total	130	100.00	70	100.0	-

The analysis of the degree of anaemia and regularity of intake of haematinic supplements showed that 104 participants (80%) in the mild anaemia group were not regularly taking haematinic supplements during pregnancy, whereas 26 participants (20%) were taking haematinic supplements daily. On the other hand, 66 (94.3%) participants in the moderate or severe anaemia group were not regularly taking haematinic supplements during pregnancy, and only 4 (5.7%) were taking them daily. The difference between the groups for severity of anaemia with regularity of haematinic supplement intake was significant (*p* = 0.026).

Analysis of the degree of anaemia with vitamin and iron levels showed that 102 participants (78.5%) in the mild anaemia group had low serum iron, 76 participants (58.5%) had low folate and 10 participants (7.9%) had low vitamin B12, while 53 participants (88.4%) in the moderate anaemia group had low serum iron, 23 (38.4%) participants had low red cell folate and 8 (13.3%) participants had low vitamin B12. However, 8 participants (80%) in the severe anaemia group had a low iron profile, 7 (70%) had low red cell folate, and all had normal levels of vitamin B12. The difference between the two groups was significant (*p* = 0.031). On the other hand, neither RBC folate nor vitamin B12 levels showed significant differences with the degree of anaemia (*p* = 0.222 for RBC folate level and *p* = 0.112 for B12 level) (Table 5).

**TABLE 5:** Differences in the degree of anaemia with iron and vitamin levels, Sudan, September 2016 to February 2017.

Characteristics	Mild anaemia		Moderate anaemia		Severe anaemia		*p*-value
	*N*	%	*N*	%	*N*	%	
**Iron study**							
Normal	28	21.5	7	11.6	2	20.0	0.031
Low	102	78.5	53	88.4	8	80.0	-
Total	130	100.00	60	100.0	10	100.0	-
**Red cell folate**							
Normal	54	41.5	37	61.6	3	30.0	0.222
Low	76	58.5	23	38.4	10	70.0	-
Total	130	100.00	60	100.0	10	100.0	-
**Serum B12**							
Normal	120	92.3	52	86.7	10	100.0	0.112
Low	10	7.7	8	13.3	0	0.0	-
Total	130	100.00	60	100.0	10	100.0	-

## Discussion

Generally, the study participants had low socio-economic status, which agrees with what was stated by Nirmala et al.,^8^ and Zaheer et al. in India.^9^ Also, 39% of the entire group were illiterate; this was consistent with findings of Melku et al. from Ethiopia,^5^ but it was a little higher than reported in another Ethiopian study by Lelissa et al.^10^ Twenty-five per cent of the participants were divorced or separated, which is consistent with what was reported in India.^11^ These findings suggest that socio-economic status could be an early predictor or an indication of anaemia among pregnant women in Sudan.

Additionally, an increasing prevalence of high parity among participants was observed (82% of the participants were multigravidas or grand multiparas). Similarly, 78% of the participants were in late pregnancy (third trimester). This observation is consistent with the findings of Amardeep et al.^12^ It indicates that the higher the parity, or the more advanced the pregnancy, the higher the likelihood of having anaemia; however, it can be explained by the depletion of mineral and vitamin stores in repeated pregnancies and increasing demands of late pregnancy.

Morphological patterns of anaemia in pregnancy seem substantially diverse across the globe, because of the diversity in aetiological factors. This study showed 58% of participants with a dimorphic (microcytic and macrocytic) pattern, followed by 25% of participants with a microcytic hypochromic pattern, 10% of participants with a macrocytic pattern and 7% of participants with a normocytic normochromic pattern. Low socio-economic status and multi-nutritional deficiency (based on low vitamin and iron levels) in the participants may explain the increased prevalence of the dimorphic pattern among them; comparable results were found in Malawi^13^ and Palestine.^14^ Surprisingly, Melku et al. in neighbouring Ethiopia reported a lower prevalence of the dimorphic pattern (4%) among pregnant women.^5^ However, results from Rajasthan state in India showed the microcytic hypochromic pattern to be the most frequent (51%), followed by normocytic normochromic (32%), dimorphic (13%) and macrocytic (4%).^15^ These results were also supported by a further study from Maharashtra state in India, which demonstrated microcytic hypochromic as the most common pattern in pregnancy (55.4%);^16^ the latter findings can be attributed to the fact that most of the Indian participants were vegetarian, and the tendency to develop iron anaemia rather than mixed deficiency anaemia is greater in this population.

Although Amardeep et al., in a study conducted in a rural population of central India, postulated a causal relationship in which iron deficiency results from folate deficiency,^12^ we believe the association is linked to the coexistence of a lack of folate and iron in the participants’ diet. This study demonstrated that the dimorphic pattern is tightly associated with low levels of both iron and RBC folate; therefore, it is assumed that the presence of a dimorphic pattern could be used as an early predictor of deficiency or as a first clue for empirical haematinic therapy in countries where full investigation is unavailable or expensive. Similarly, the microcytic hypochromic pattern was strongly related to a low iron profile and hence can be an indicator for empirical iron therapy in the management of anaemia. However, empirical administration of folic acid to anaemic patients with a macrocytic or dimorphic (microcytic or macrocytic) pattern could potentially result in undesirable consequences such as neuropathy due to excessive use of vitamin B12 to facilitate entering of serum folate to the cells.^5^ On the other hand, our results showed that the dimorphic pattern tended to present as mild anaemia (64%), whereas the microcytic hypochromic pattern presents as moderate or severe anaemia, which agrees with what was reported by Nirmala et al.^8^ These findings are contradictory to the postulations that the more severe the anaemia, the more likely it is to be a result of multiple deficiencies and not related solely to iron deficiency.^12^

Moreover, a relatively high prevalence of the dimorphic pattern (88%) among those who did not regularly take haematinic supplements was observed and can be attributed to the lack of a national programme for providing free haematinic supplements to pregnant Sudanese women. Also, our results showed an increase in the dimorphic pattern in late pregnancy: 88% of the dimorphic pattern group was in the third trimester, a finding consistent with what was reported by Nirmala et al.^8^

Our study demonstrated a low percentage of the microcytic hypochromic pattern (25%) compared with what was reported by Babu et al. in India (64%) and Melku et al. in Ethiopia (60%).^5,11^ This finding could be attributed to diversity in nutritional habits across these countries, for example, excessive drinking of iron inhibitors in tea and coffee in Ethiopia or being vegetarian in India, or because of insufficient intake of foods or drinks rich in vitamin C, such as citrus fruits, and low bioavailability of dietary iron. Although thalassemia contributes to the prevalence of microcytic hypochromic anaemia in the area, the finding of a low iron profile in our participants suggests that iron deficiency rather than thalassemia was the cause of the microcytic hypochromic pattern. On the other hand, the microcytic hypochromic pattern was highly prevalent among multigravidas, which is consistent with what was reported by Amardeep et al.,^12^ and could be explained by the depletion of iron stores over repeated pregnancies.

Ten per cent of participants had the macrocytic pattern, 80% of them had low RBC folate and 70% had low vitamin B12; the majority of participants with this pattern had both vitamin deficiencies. Thus, it is difficult to interpret the coexistence of low levels of both vitamins without considering the malabsorption state in pregnancy. Generally, folate deficiency is more common than vitamin B12 deficiency in pregnancy, because increasing demands in pregnancy causes folate deficiency more than vitamin B12, whereas the latter results more commonly from malabsorption. However, both can be worsened by poor diet and low intake of milk and vegetables.^12^

The normocytic normochromic pattern was the least frequent; only 7% of participants presented with this pattern, and all of them had average vitamin and iron levels. Although the study was not designed to detect the exact aetiology, parasitic infestation or micro-mineral deficiency (e.g. zinc and copper) could be involved. This postulation was proposed by two different studies conducted in Sudan^17^ and Malawi.^13^ Several studies from around the globe suggest that deficiencies of other haematinics and chronic disease can contribute to the normocytic normochromic pattern. Indonesian research assumed that lack of vitamin A might help cause the normocytic normochromic pattern in pregnancy.^18^ Our study revealed that all participants exhibiting this pattern had average levels of iron, vitamin B12, and folate, and were taking haematinic supplements daily during pregnancy. Therefore, we assumed microelements might be the cause, as chronically ill patients were excluded from the study.

On the other hand, our study demonstrated statistically significant differences in the degree of anaemia across parity, gestational age and haematinic supplement intake; that is, increased parity, later gestational age and lack of regular haematinic supplement intake were associated with increased prevalence of severe anaemia. These findings were consistent with those of Shankar et al.^2^ and Al-Farsi et al.^19^ Additionally, the degree of anaemia revealed statistically significant differences with iron levels; that is, severe anaemia substantially increased with a low iron profile, but not with RBC folate or with vitamin B12 levels. These findings may explain why the dimorphic pattern tended to present as mild anaemia, whereas the microcytic hypochromic pattern presented as moderate or severe anaemia.

### Limitations

The most crucial weakness in the study was that it was conducted in one hospital in Khartoum city; stronger results would be obtained if it was a multicentre study. Moreover, the setting and design of the study were not sufficient to detect the exact aetiology of the morphological pattern.

### Recommendations

We recommend using RBC morphology for accurate diagnosis, management and follow-up of anaemia during pregnancy in all primary healthcare centres in Sudan (at least, doing a peripheral smear for morphology). Also, we recommend doing further studies on the possibility of using the morphological pattern to predict folate and iron deficiency and hence how and what haematinic supplements should be used in areas like Sudan where a full investigation is not available. Further studies on the topic are recommended in developing countries.

### Conclusion

The most frequent morphological pattern of anaemia among the pregnant women in this study was the dimorphic (mixed) pattern, followed by microcytic hypochromic, macrocytic and normochromic normocytic, which is in line with evidence for global diversity in the frequency of morphological patterns of anaemia in pregnancy. Morphological patterns may predict the type of vitamins and mineral deficiency and we found some evidence to support this as a possibility; that is, the dimorphic pattern may be a predictor of mixed folate and iron deficiency, whereas the microcytic hypochromic pattern may be a predictor of iron deficiency during pregnancy. Moreover, morphological patterns may predict the degree of anaemia as the dimorphic pattern tended to present as mild anaemia, whereas the microcytic hypochromic presented as moderate or severe anaemia. The study also concluded that there are significant differences in the severity of anaemia with parity, gestational age and lack of regular haematinic supplement intake.
